# Evolutionary morphology of podocytes and primary urine-producing apparatus

**DOI:** 10.1007/s12565-015-0317-7

**Published:** 2015-12-01

**Authors:** Koichiro Ichimura, Tatsuo Sakai

**Affiliations:** Department of Anatomy and Life Structure, Juntendo University Graduate School of Medicine, 2-1-1 Hongo, Bunkyo-ku, Tokyo, 113-8421 Japan

**Keywords:** Protonephridium, Metanephridium, Glomerulus, Terminal cell, Podocyte

## Abstract

Excretory organs were acquired in the early phase of metazoan evolution, and they play a crucial role in the maintenance of homeostasis of body fluids. In general, these organs consist of two functional components, the primary-urine producing apparatus and the modulating tubule. This basic organization of the excretory organs is conserved among most metazoans. Herein, we present an overview of the morphological evolution of the primary urine-producing apparatus in metazoans and describe the acquisition of the renal glomerulus—a specialized primary urine-producing apparatus—in vertebrates. We also describe the advancement of the glomerular structure and function in higher vertebrates.

## Introduction

The excretory organ is a viscus essential in maintaining homeostasis in almost all metazoans. There is wide structural diversity among metazoan excretory organs, but they are commonly divided into two functional compartments: the primary urine-producing apparatus (PUPA), and the modulating tubule. For instance, in vertebrate kidneys, the glomerulus and renal tubule are homologous to the PUPA and modulating tubule, respectively. In general, the PUPA is formed mainly from epithelial cells such as terminal cells and podocytes, which are highly specialized for ultrafiltration of body fluids. The primary urine is produced from body fluids (interstitial fluid or blood plasma) by ultrafiltration in most metazoan taxa. The primary urine is subsequently transferred to the modulating tubule, where it is modified by epithelial secretion and reabsorption and discharged outside the body as final urine. In this review, we summarize the morphological evolution and diversity of PUPA and filtration epithelial cells in metazoans. In particular, we focus on the acquisition of the renal glomerulus—a type of PUPA unique to vertebrates—and the structural advancement of glomerulus and podocytes in higher vertebrates.

### Terminal cell-based PUPA in protonephridia

**Fig. 1a,b:**
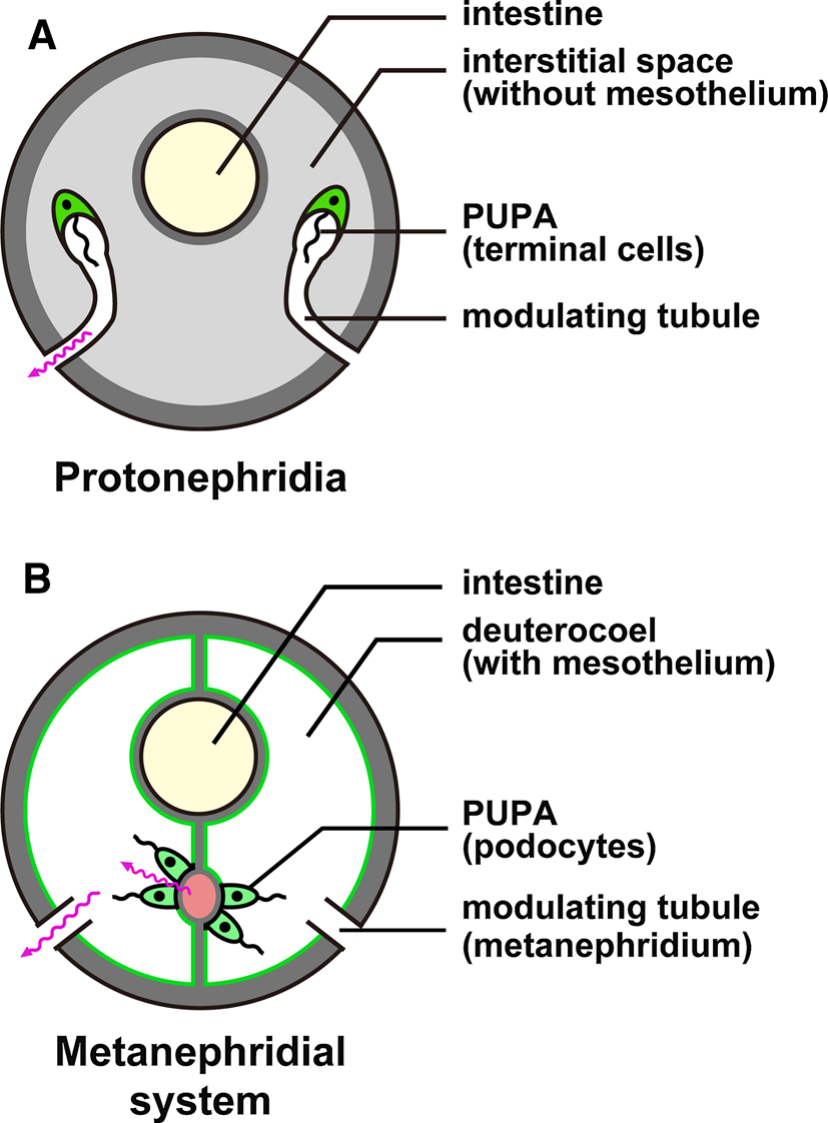
Two types of excretory system in metazoans. **a** Protonephridia. The primary urine-producing apparatus (PUPA) is formed by terminal cells and is connected directly to the modulating tubule. Protonephridia are found in the interstitial space. **b** Metanephridial system. The metanephridium contains only the modulating tubule. A part of the mesothelium is altered into podocytes to form the PUPA. The term “metanephridial system” is used to indicate the assemblage of the podocyte-based PUPA and metanephridium. These schematic drawings are based on Ruppert and Smith (1988)

The morphology of an excretory organ is related largely to whether the animal possesses a deuterocoel, which is a body cavity lined with a simple epithelial sheet called mesothelium (Ruppert et al. 2003; Ruppert and Smith 1988). In general, animals without the deuterocoel (so-called acoelomates and pseudocoelomates) possess protonephridium as excretory organ (Fig. 1a), whereas animals with the deuterocoel (eucoelomates including vertebrates) have metanephridium (Fig. 1b). However, some eucoelomate groups possess both types of excretory organs.

The protonephridium is an epithelial tubular system, whose proximal blind end serves as a PUPA formed by filtration epithelial cells—the terminal cells. The terminal cells are extremely variable in structure among animal taxa and are known as flame cells, cyrtocytes, and solenocytes (Wilson and Webster 1974). The remaining region of protonephridium is a modulating tubule, whose distal end opens outside the body to discharge the final urine. A number of motile cilia are found on the luminal surface of both the terminal cells and epithelial cells of modulating tubule. Continuous movement of the motile cilia transfers the primary urine toward the distal end, producing the transmural pressure gradient necessary for filtration between the interstitial space and protonephridial lumen.

**Fig. 2a–c:**
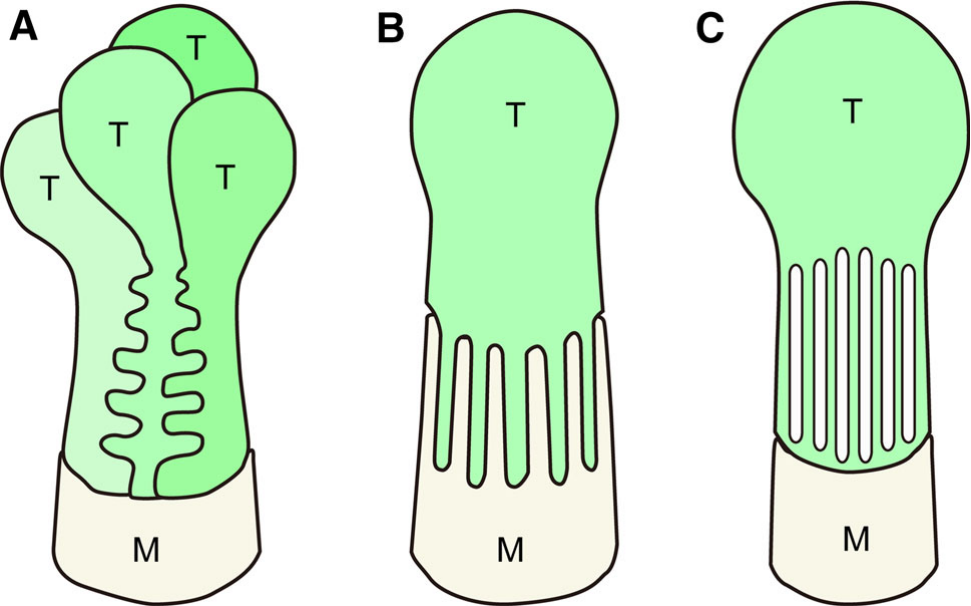
Types of terminal cells in protonephridia. Terminal cells are broadly divided into several types. In most species, the filtration slits are an intercellular space between the terminal cells (**a**) or between the terminal cell and modulating tubular epithelial cell (**b**). In some animals, numerous rectangular filtration slits/fenestrations are opened at the tubular cytoplasmic wall of the terminal cell (**c**).* T* Terminal cell,* M* epithelial cell of modulating tubule. These schematic drawings are based on Kieneke et al. (2008)

**Fig. 3a–d:**
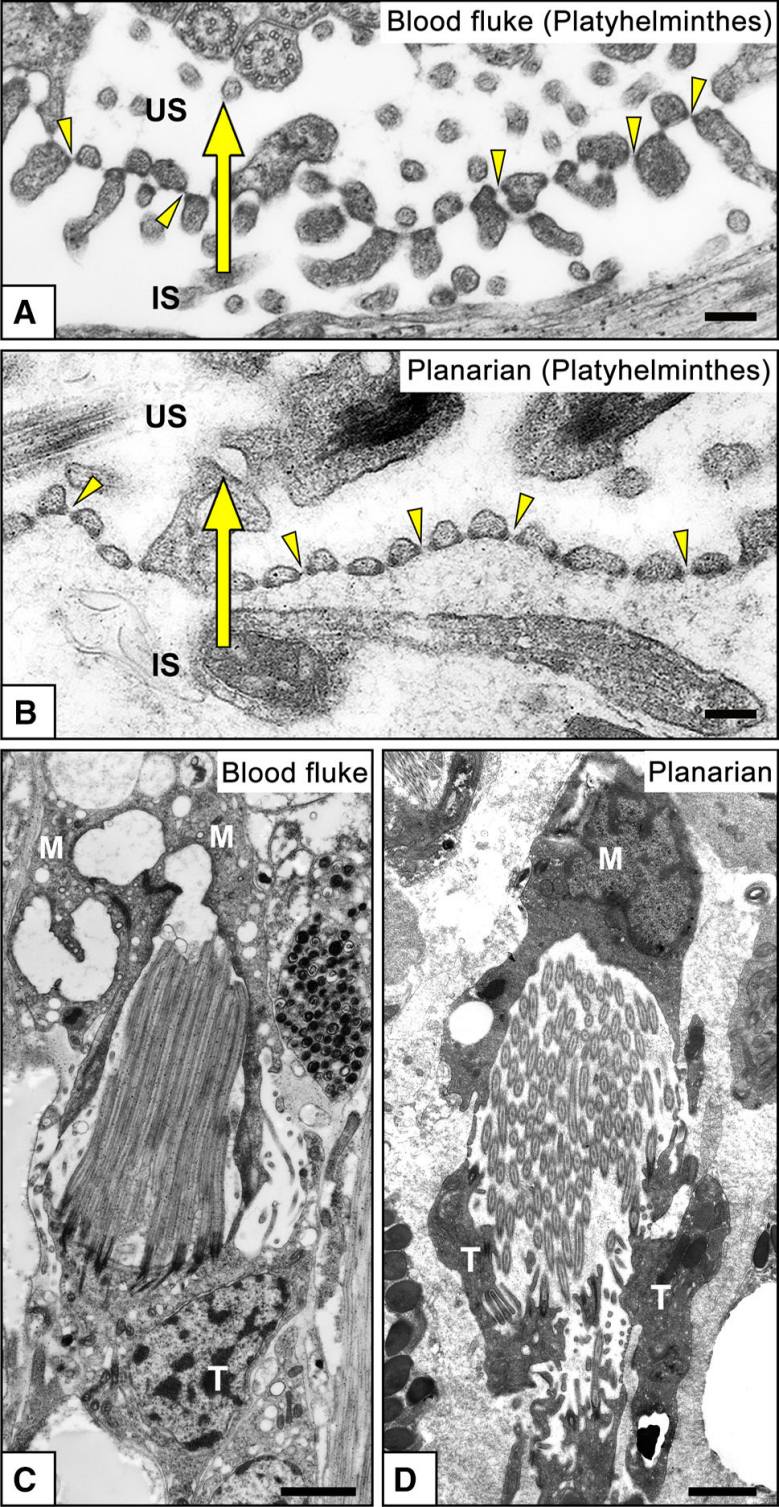
Terminal cells in Platyhelminthes. Transmission electron micrographs of terminal cells in the blood fluke *Schistosoma mansoni* (**a**, **c**), and freshwater planarian *Dugesia japonica* (**b**, **d**). Terminal cells form filtration slits for ultrafiltration, which are spanned by membranous structures, the slit diaphragms (*arrowheads* in **a**, **b**). The attachment sites of slit diaphragms are associated with electron-dense plaques. The terminal cell is interdigitated with a modulating tubular epithelial cell to form filtration slits in the blood fluke (as shown in Fig. 2b). In the planarian, formation of the filtration slits remains unclear. Several dozen motile cilia protrude from the luminal surface of the terminal cell (**c**, **d**).* IS* Interstitial space,* M* epithelial cell of modulating tubule,* T* terminal cell,* US* urinary space (lumen of protonephridium). *Arrows* Direction of primary urine flow. *Bars* **a**, **b** 200 nm; **c**, **d** 5 μm

In most of the protonephridial PUPA, the primary urine passes through a filtration slit, an intercellular space that forms between the terminal cells (Fig. 2a), or between the terminal and modulating tubular epithelial cells (Fig. 2b) (Kieneke et al. 2008; Wilson and Webster 1974). In some animals, the terminal cell is shaped like a test tube with numerous rectangular filtration slits (or fenestrations) on its cytoplasmic wall (Fig. 2c). In the three types of terminal cell, the filtration slit has a constant width and is bridged by a membranous structure (Fig. 3a, b) that is similar to the slit diaphragm found in podocytes, the metanephridial filtration epithelial cells (see next section). For the sake of convenience, we refer to this membranous structure in the terminal cell as the slit diaphragm. Analogous to vertebrate podocytes, the slit diaphragm in terminal cells, together with the basement membrane, prevents the leakage of macromolecules from body fluid into the primary urine.

A three-dimensional structure of terminal cells has been suggested in a number of species, but in some species this structure needs further reevaluation. For example, in freshwater planarian, Ishii (1980) suggested that the filtration slits are formed between terminal cells (Fig. 2a), whereas another study reported that the filtration slits are formed as numerous rectangular fenestrations that open onto the tubular cytoplasmic wall of terminal cells (Fig. 2c) (McKanna 1968).

### Podocyte-based PUPA in invertebrate metanephridial systems

**Fig. 4a–k:**
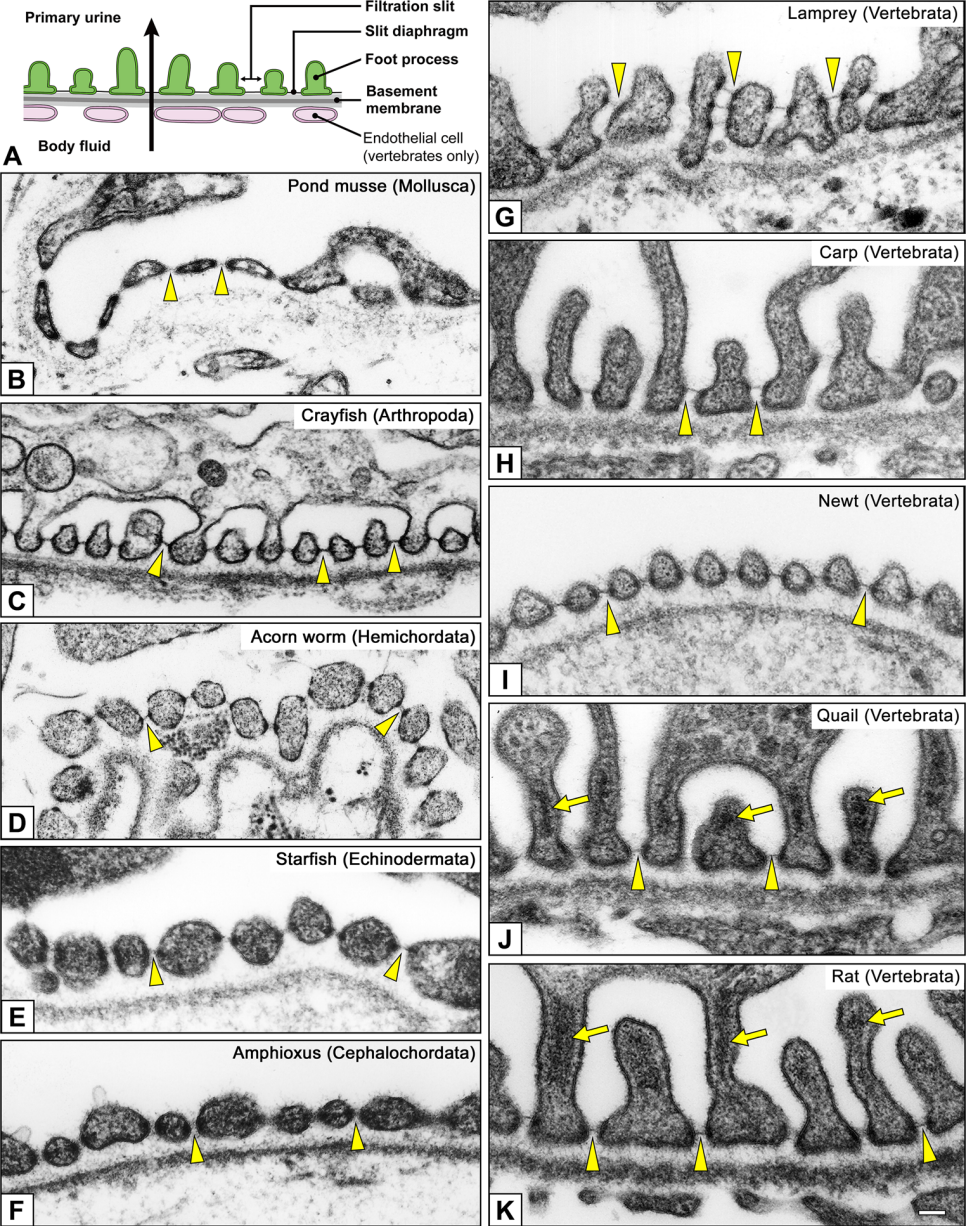
Foot processes and slit diaphragms in podocytes. Basic structure of the metanephridial podocytes is conserved among eucoelomates. The filtration site consists of the foot processes, slit diaphragms, and basement membrane (**a**); the foot processes widely vary in shape and size among invertebrates (**b**–**f**) and vertebrates (**g**–**k**). The foot processes contain prominent actin bundles only in quail and rat podocytes (*arrows* in **j**, **k**). Actin bundles contribute to the mechanical protection of the glomerular wall from higher glomerular capillary pressure peculiar to birds and mammals. In vertebrates, glomerular endothelial cells are also contained in the filtration barrier. **b** Pond mussel (*Sinanodonta lauta*), **c** Crayfish (*Procambarus clarkii*), **d** Acorn worm (*Balanoglossus misakiensis*), **e** Starfish (*Patiria pectinifera*), **f** Amphioxus (*Branchiostoma japonicum*), **g** Lamprey (*Lampetra japonica*), **h** Carp (*Cyprinus carpio*), **i** Newt (*Cynops pyrrhogaster)*, **j** Quail (*Coturnix japonica*), **k** Rat (*Rattus norvegicus*). *Arrowheads* slit diaphragm. *Bar * 200 nm

**Fig. 5a–f:**
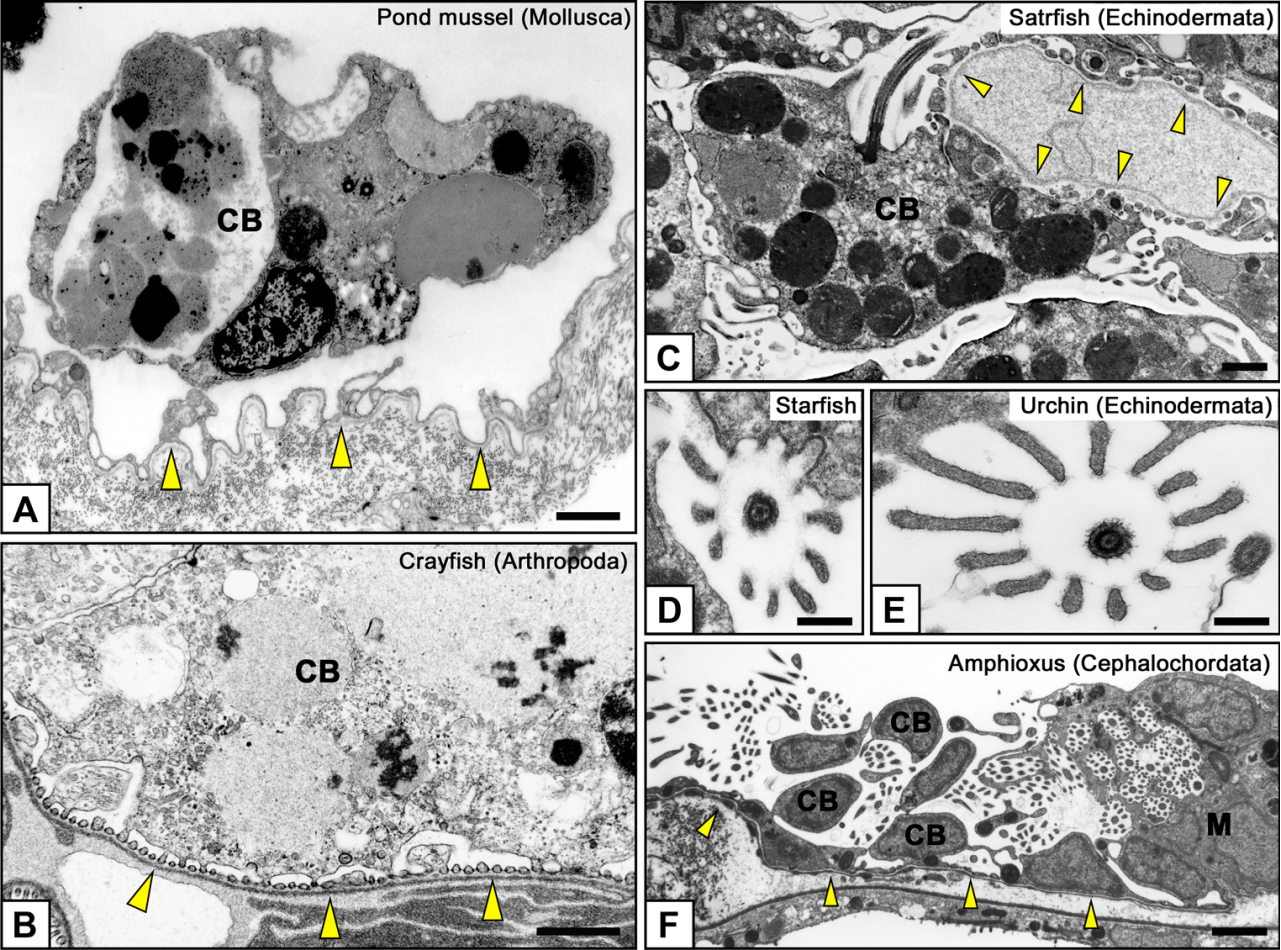
Characteristic structures of podocytes in invertebrates. Well-developed lysosomes are frequently found within the podocyte cell bodies in invertebrates (**a**–**c**), suggesting that the podocytes may play a role in the isolation of non-excreted toxic substances to maintain the quality of hemolymph in invertebrates. Podocytes generally possess a single motile cilium on the cell body. In echinoderms and cephalochordates, the motile cilium is protruded together with a microvillus collar, which consists of 10–12 microvilli and surrounds a motile cilium in a circle (**d**–**f**). The motile cilium with microvillus collar enters the metanephridium from the nephrostome in cephalochordates and is highly likely to lead the primary urine from the body cavity to metanephridium (**f**). **a** Pond mussel, **b** Crayfish, **c**, **d** Starfish, **e** Sea urchin (*Hemicentrotus pulcherrimus*), **f** Amphioxus. *Arrowheads* basement membrane of podocytes.* CB* Cell body of podocyte,* M* metanephridium. *Bars* **a–c** 1 μm; **d**, **e** 200 nm; **f** 5 μm

In most eucoelomates, the metanephridium is used as an excretory organ instead of, or in addition to, the protonephridium. The protonephridium contains both the PUPA and modulating tubule, while the metanephridium contains only the modulating tubule. To form a PUPA, most eucoelomates alter the mesothelial cells at specific sites into podocytes, the filtration epithelial cells (Figs. 1b, 4, 5). In some groups of eucoelomic invertebrates (annelid, molluscs, and others), the mesothelial cells associated with large vasculatures and the heart are altered into podocytes, which presumably improves the efficiency of filtration. The primary urine is temporally stored in the coelomic cavity, and then enters the metanephridium through its proximal opening, metanephridial funnel, or nephrostome. The metanephridial funnel possesses a number of motile cilia whose movement contributes to the aspiration of primary urine into the metanephridium. As proposed by Ruppert and Smith (1988), the term “metanephridial system” is also used to correlate the podocyte-based PUPA and metanephridium.

Unlike terminal cells, the basic cytoarchitecture of podocytes is conserved among animal species (Ruppert et al. 2003; Takahashi-Iwanaga 2002). Podocytes consist of three subcellular compartments: the cell body, primary processes, and foot processes (Kriz and Kaissling 2007; Mundel and Kriz 1995). Podocytes interdigitate with each other to form numerous foot processes, by which they adhere to the basement membrane (basal lamina) (Fig. 4). The foot processes protrude from the podocyte’s cell body and primary processes that extend from the cell body. In some invertebrates, the primary processes are not developed and podocytes seem to consist only of the cell body and foot processes.

Between foot processes of neighboring podocytes, an intercellular space—the filtration slit—is maintained for the passage of primary urine. The filtration slit is spanned by the slit diaphragm—a membranous intercellular junction specialized for ultrafiltration. In vertebrate podocytes, the slit diaphragms, together with their basement membrane, are known to play a role in the maintenance of selective filtration barrier, which prevents macromolecules in blood plasma from leaking into the primary urine (Kramer-Zucker et al. 2005; Pavenstadt et al. 2003). In invertebrate podocytes, the slit diaphragm very likely contributes to the maintenance of the selective filtration barrier, but its functional properties have not been elucidated.

In general, invertebrate podocytes exhibit extremely developed lysosomes, which frequently contain crystal or amorphous materials (Fig. 5a–c), suggesting that the podocytes may play a role in the isolation of non-excreted toxic substances from hemolymph to maintain its quality in invertebrates. Such developed lysosomes were not found in the intact podocytes of vertebrates.

**Fig. 6:**
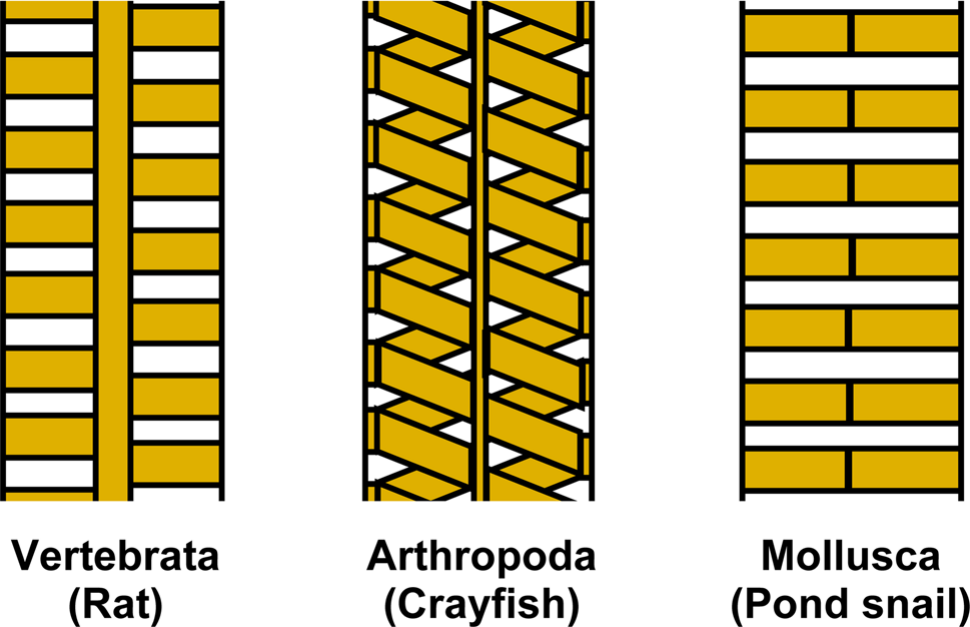
Structural differences in podocyte slit diaphragm. An ultrastructural model of slit diaphragm was proposed in three kinds of eucoelomates on the basis of previous research using en bloc staining with tannic acid (Boer and Sminia 1976; Rodewald and Karnovsky 1974; Schaffner and Rodewald 1978). *Yellow* Framework of slit diaphragm, *white* interstices, which is the passage for primary urine. These models illustrate the wide structural differences in the podocyte slit diaphragms among metazoan species (color figure online)

The structure of the podocyte slit diaphragm has been investigated in several types of animals using scanning electron microscopy (SEM), transmission electron microscopy (TEM), and TEM tomography (Gagliardini et al. 2010; Rodewald and Karnovsky 1974; Wartiovaara et al. 2004). TEM with tannic acid-staining indicated the structural differences of slit diaphragms among rat, crayfish, and freshwater snail (Boer and Sminia 1976; Rodewald and Karnovsky 1974; Schaffner and Rodewald 1978) (Fig. 6). The structural differences imply that the molecular components of the slit diaphragm are also different among animals. The slit diaphragm in vertebrate podocytes is known to be a complex of several dozen membrane and intracellular proteins. In particular, three kinds of membrane proteins are essential for building and maintaining the slit diaphragm in vertebrate podocytes: nephrin, podocin, and neph1 (Boute et al. 2000; Ichimura et al. 2013; Kestila et al. 1998; Kramer-Zucker et al. 2005). However, the molecular components of slit diaphragm remain unknown in invertebrate podocytes.

Podocytes possess one cilium per each cell, whose structure differs greatly between invertebrates and vertebrates. Invertebrate podocytes frequently comprise a motile cilium with 9 + 2 axoneme (Fig. 5d–f). In echinoderms and amphioxus, the motile cilium protrudes together with a microvillus collar, which consists of 10–12 microvilli and surrounds a motile cilium in a circle (Fig. 5d, e). In amphioxus, the motile cilium with microvillus collar enters the metanephridium from the nephrostome and is likely to lead the primary urine from the body cavity to the metanephridium. However, the same structure in echinoderms does not link to the metanephridium and its function remains unclear. Unlike invertebrate podocytes, vertebrate podocytes have a primary (non-motile) cilium with 9 + 0 axoneme (Ichimura et al. 2010; Miyoshi 1978; Ojeda et al. 2003; Zuasti et al. 1983). The primary cilia are cellular mechano-sensors that are responsible for the sensation of fluid flow (Praetorius 2015; Praetorius and Spring 2003). Functional abnormality of primary cilium-related proteins disturbs the planar cell polarity in renal tubules, which causes cystic kidney disease (Fedeles and Gallagher 2013). However, the function of primary cilia is not clearly understood in podocytes.

### Nephrocyte, a podocyte-related cell in invertebrates

**Fig. 7a–d:**
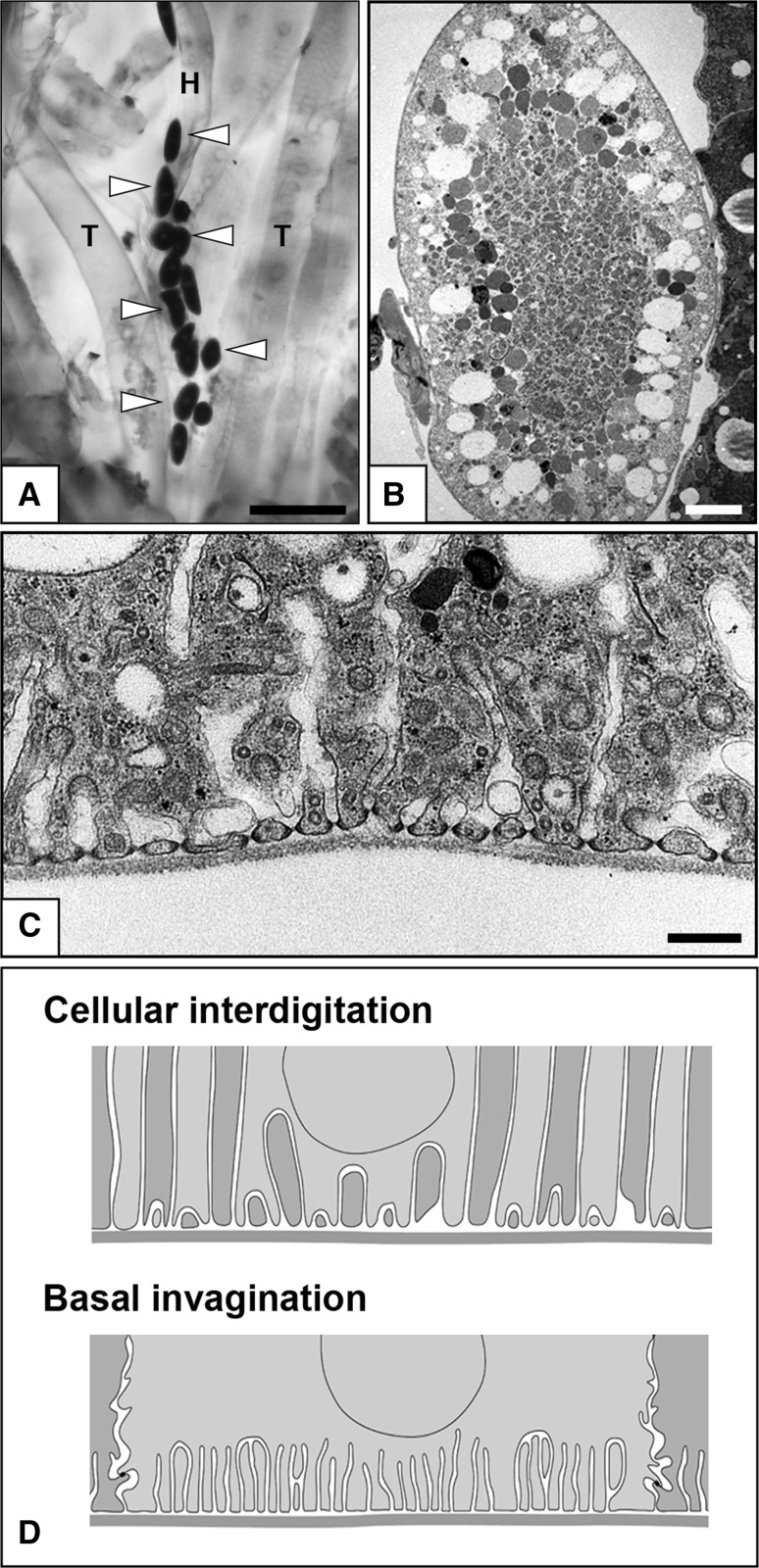
Nephrocytes in the fruit fly, *Drosophila melanogaster*. **a** Nephrocytes are detected by in situ hybridization for *cathepsin B1* mRNA in larval fruit fly (*arrowheads*), and they are localized beside the heart tube. *H* Heart tube, *T* trachea. **b**, **c** Transmission electron micrographs of nephrocytes. Abundant endocytotic vesicles and well-developed lysosomes are characteristic in nephrocytes, which are crucial for the quality control of hemolymph. Nephrocytes protrude numerous foot processes adhering to the basement membrane and possess a slit diaphragm between the foot processes (**c**). **d** Comparison of cellular interdigitation and basal invagination in epithelial cells. The foot processes are formed by cellular interdigitation in podocytes and invagination of basal plasma membrane in nephrocytes. Thus, the slit diaphragm is an intercellular junction in podocytes and an autocellular junction in nephrocytes. *Bars* **a** 50 μm, **b** 5 μm, **c** 200 nm

In several phyla of eucoelomates (Arthropoda, Onycophora, and Mollusca), nephrocytes, which are similar to podocytes in structure, are found in the interstitial or hemolymph spaces, and they are not associated with the PUPA of the metanephridial system (Boer and Sminia 1976; Goodman and Cavey 1990; Kokkinopoulou et al. 2014; Ruppert 1994; Seifert and Rosenberg 1977) (Fig. 7). A single or several nephrocytes are completely surrounded by the basement membrane, which is produced by nephrocytes themselves. The nephrocytes are organized into numerous foot processes adhering to the basement membrane and possess a slit diaphragm between the foot processes (Fig. 7c). There is no direct connection between the nephrocytes and modulating tubule (metanephridium and Malpighian tubule); thus, the nephrocytes do not serve as a PUPA. Instead, the nephrocytes are known to play a crucial role in the isolation of toxic substances, such as heavy metals, from hemolymph (Zhang et al. 2013a). Abundant endocytotic vesicles and highly developed lysosomes exist in the nephrocytes, reflecting the functional property of the cells (Fig. 7b, c).

The foot processes are quite similar in structure between nephrocytes and podocytes. However, formation of the foot processes is very different. As mentioned above, the podocyte foot processes are formed by the interdigitation between neighboring podocytes, and thus the podocyte slit diaphragm becomes one of the intercellular junctions (Fig. 7d). In contrast, in nephrocytes, the foot processes are formed by invagination of the basal plasma membrane, and the nephrocyte slit diaphragm is regarded as an autocellular junction (Fig. 7d). In spite of this structural difference, the molecular components of the slit diaphragm are highly conserved between the nephrocytes in fruit fly (*Drosophila melanogaster*) and podocytes in vertebrates (Weavers et al. 2009; Zhuang et al. 2009). Moreover, nephrocyte-specific gene knockdown can be performed easily using GAL4/UAS RNA interference in *D. melanogaster* (Zhang et al. 2013b), making them an attractive novel model system for research of molecular functions of podocyte-specific proteins.

### Unique excretory systems producing primary urine by active transport

In most metazoan excretory systems, the primary urine is produced by ultrafiltration in the protonephridial and/or metanephridial system. Several other kinds of excretory systems possess no filtration epithelium and produce primary urine by epithelial active transport of the modulating tubule. The Malpighian tubule is a characteristic excretory system in insects and tardigrades; the proximal end is blind and distal end opens into the intestine. This tubular system lacks filtration epithelial cells and has no continuity with the above-mentioned nephrocytes (Beyenbach et al. 2010; Singh and Hou 2008). Nematodes also have two unique excretory systems, an excretory gland (renette cell or ventral cell) and excretory canal (H canal), both of which open at a midventral point of the body surface (Turpenniemi and Hyvarinen 1996). Like the Malpighian tubule, these two systems contain no filtration epithelial cells and produce urine only by epithelial active transport.

As described in the next section, most vertebrates utilize the glomerulus in the kidney nephron as a PUPA. Exceptionally, some marine teleost fish lack a glomerulus and produce urine only by active transport of proximal tubular epithelial cells (such fish are called “aglomerular teleost fish”) (McDonald and Grosell 2006). It remains unknown whether the glomeruli never form during nephrogenesis, or if they are formed initially but degenerate later.

### Acquisition and evolutionary development of renal glomerulus in vertebrates

**Fig. 8a–e:**
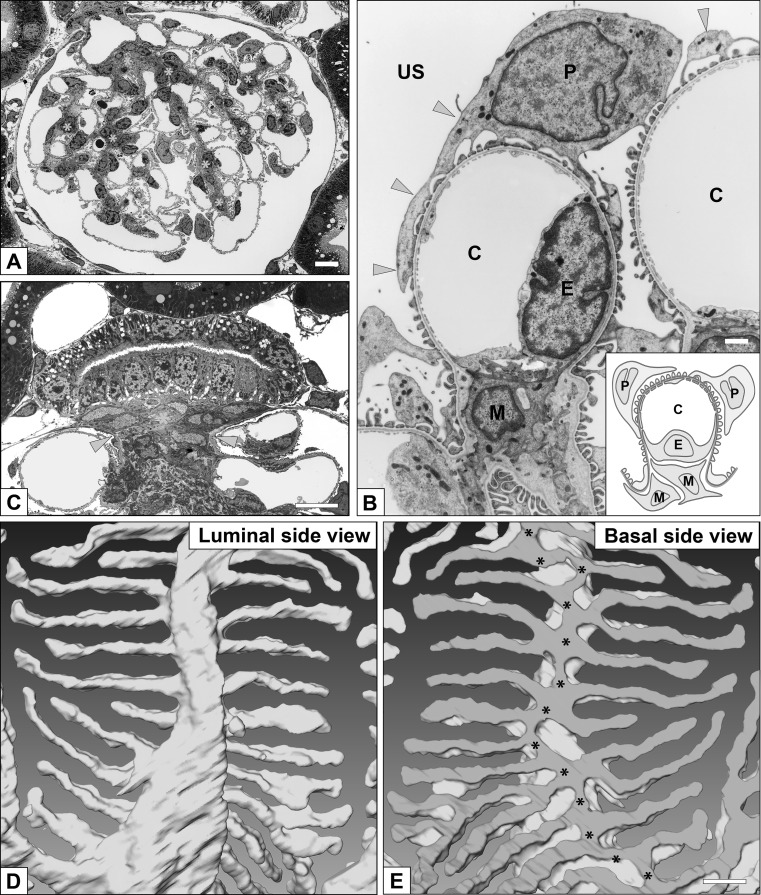
Glomerulus and podocytes in mammalian metanephros. **a** Renal glomerulus contains capillary loops (*pale pink*) and mesangium (*asterisks*). **b** Three cellular components of glomerulus. Podocytes (P) and glomerular basement membrane covers the glomerular capillary, made by fenestrated endothelial cells (E), and mesangium containing mesangial cell (M) and matrix. C, capillary lumen. Podocytes consist of three subcellular compartments: cell body, primary processes (*arrowheads*), and foot processes. **c** The extraglomerular mesangium (*asterisk*) occupies the space surrounded by the macula densa and glomerular arterioles as a tectum for sealing the glomerular vascular hilum in mammals. The extraglomerular mesangium is composed of flat-shaped extraglomerular mesangial cells and a small amount of extracellular matrix surrounding these cells. **d**, **e** Three-dimensional structure of podocyte foot processes. A single reconstructed podocyte based on serial sectional images is observed from luminal (**d**) and basal (**e**) sides. In the basal surface view, the most proximal portions of the foot processes are connected via a tortuous ridge-like prominence (*asterisks* in **e**), which was formed on the basal surface of the primary process (see details in Ichimura et al. 2015). **a**, **b**, **d**, **e** Rat (Wistar strain); **c** House musk shrew, *Suncus murinus* (KAT strain). *Bars* **a**, **c** 5 μm; **b** 1 μm; **e** 200 nm (color figure online)

The vertebrate kidney/nephron is a type of metanephridial system that contains the glomerulus as a podocytes-based PUPA. The glomerulus has several unique features that are not seen in other metanephridial PUPA. One is the presence of glomerular capillary loops, which are formed by fenestrated endothelial cells (Ichimura et al. 2008) (Fig. 8a, b). In some invertebrates, the mesothelial cells associated with vasculature and heart are altered into podocytes to form PUPA, but the endothelial cells are not associated with the basement membrane of podocytes as found in vertebrates. However, in hemichordates, a glomerulus-like PUPA is found in the proboscis coelom and is simply called “glomerulus” (Balser and Ruppert 1990; Mayer and Bartolomaeus 2003). The vascular system in the glomerulus-like PUPA appears similar to the glomerular capillary; however, it lacks endothelial cells. Other than endothelial cells, the vertebrate glomerulus contains a characteristic third type of cell, the mesangial cell, which is a highly specialized vascular smooth muscle cell that maintains the three-dimensional architecture of glomerular capillary loops (Ichimura et al. 2006; Sakai and Kriz 1987).

During vertebrate ontogeny and phylogeny, three types of kidney (pronephros, mesonephros, and metanephros) are distinguishable on the basis of their localization and developmental process (Jollie 1973; Saxen 1987). The pronephros is one of the typical metanephridial systems. The mesothelial cells close to the dorsal aorta are altered into podocytes, which are combined with the capillary branched from the dorsal aorta to form a pronephric glomerulus. Similar to eucoelomic invertebrates, the primary urine produced by pronephric glomerulus enters the coelomic cavity and subsequently into the pronephric (modulating) tubules that open into the coelomic cavity. The pronephric tubules continue to the Wolffian duct, which terminates at the cloaca. Pronephros appears temporarily during the developmental period and gradually degenerates when the mesonephros starts to function. Finally, the pronephros disappears in most vertebrates.

The mesonephros and metanephros are also regarded as metanephridial systems, but their glomeruli are formed independently from the mesothelium (Saxen 1987). The mesonephric and metanephric nephrons are derived from mesenchymal condensations that are newly formed in the nephrogenic mesenchyme. The mesenchymal condensations are altered to renal vesicles by the mesenchymal-epithelial transition. The renal vesicle elongates and deforms in a peculiar way in each animal taxon, generating a nephron primordium whose most proximal part is altered into the Bowman’s capsule. A part of this capsule differentiates into podocytes and forms a glomerulus together with the capillary loops and mesangial cells. Formation of numerous glomeruli makes it possible to efficiently obtain a larger filtration area in the limited volume of the mesonephros and metanephros.

### Morphological evolution of glomerulus and podocyte in mammals

The vertebrate glomerulus can be divided into two structural compartments: vascular and epithelial. The vascular compartment is a core structure of the glomerulus and consists of glomerular capillary loops and mesangial cells. In contrast, the epithelial component is a sheet-like structure surrounding the vascular component en bloc; it consists of podocytes and the glomerular basement membrane (GBM). This basic architecture is highly conserved among vertebrates, although various structural modifications are found in both the vascular and epithelial compartments, especially in birds and mammals.

**Fig. 9:**
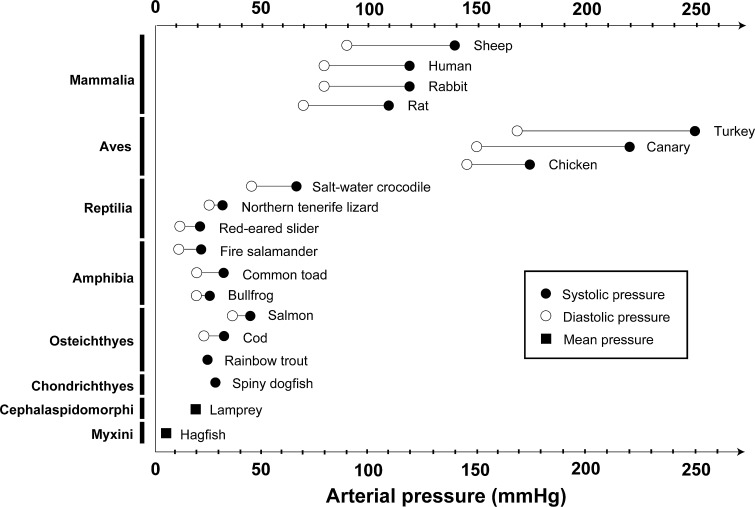
Blood pressure in vertebrates. Birds and mammals exhibit higher arterial pressure than other vertebrates. The arterial pressure data are based mainly on Prosser (1973)

Birds and mammals have acquired much higher blood pressure than other vertebrates (Braun and Dantzler 1997; Prosser 1973) (Fig. 9) and, consequently, they can maintain higher glomerular capillary pressure (GCP) to efficiently produce a large amount of primary urine. The GCP is approximately 50 mmHg in mammals (Baylis and Brenner 1978), which is about 5- to 10-fold higher than that of the aortic blood pressure in lower vertebrates. In birds, the GCP has not been reported, but considering their high blood pressure and glomerular filtration volume, it is expected to be similar to that observed in mammals. Most of the structural modifications peculiar to avian and mammalian glomeruli are closely related to their higher GCP, as described in the following paragraphs.

#### Extraglomerular mesangium

The extraglomerular mesangium (EGM) occupies the space surrounded by the macula densa of distal tubules and glomerular arterioles, as a tectum sealing the glomerular vascular hilum in mammals (Elger et al. 1998) (Fig. 8c). The EGM prevents leakage of higher GCP from the hilum and its extreme expansion. The loosening of EGM is known to lead to structural disorganization of glomerular structure, such as the wide expansion of the vascular hilum observed in angiotensin II receptor gene-null mice (Inokuchi et al. 1998).

The EGM is peculiar to the mammalian glomerulus, but a structure that corresponds functionally to the EGM has been detected in avian glomeruli. The mesangium is massive and occupies a central region of the glomerulus in birds. This massive mesangium itself is plugged into the glomerular hilum to prevent leakage of GCP in birds.

#### Well-developed stress fibers in mesangial cells

Mesangial cells possess numerous spinous cytoplasmic processes whose tips adhere to the inner surface of the GBM (Farquhar and Palade 1962; Takahashi-Iwanaga 2015). In birds and mammals, stress fibers—an actin filament-based contractile apparatus—are prominently developed within mesangial cells. Both ends of the stress fibers are inserted into mesangial cell processes and are linked to the inner surface of the GBM. The tractive force of the stress fibers prevents the glomerular epithelial compartment from overexpansion due to higher GCP. The mesangial cells in vertebrates other than birds and mammals have a fibroblast-like shape, and their cytoplasmic processes do not contain any prominent stress fibers.

#### Actin bundles in podocytes

**Fig. 10a,b:**
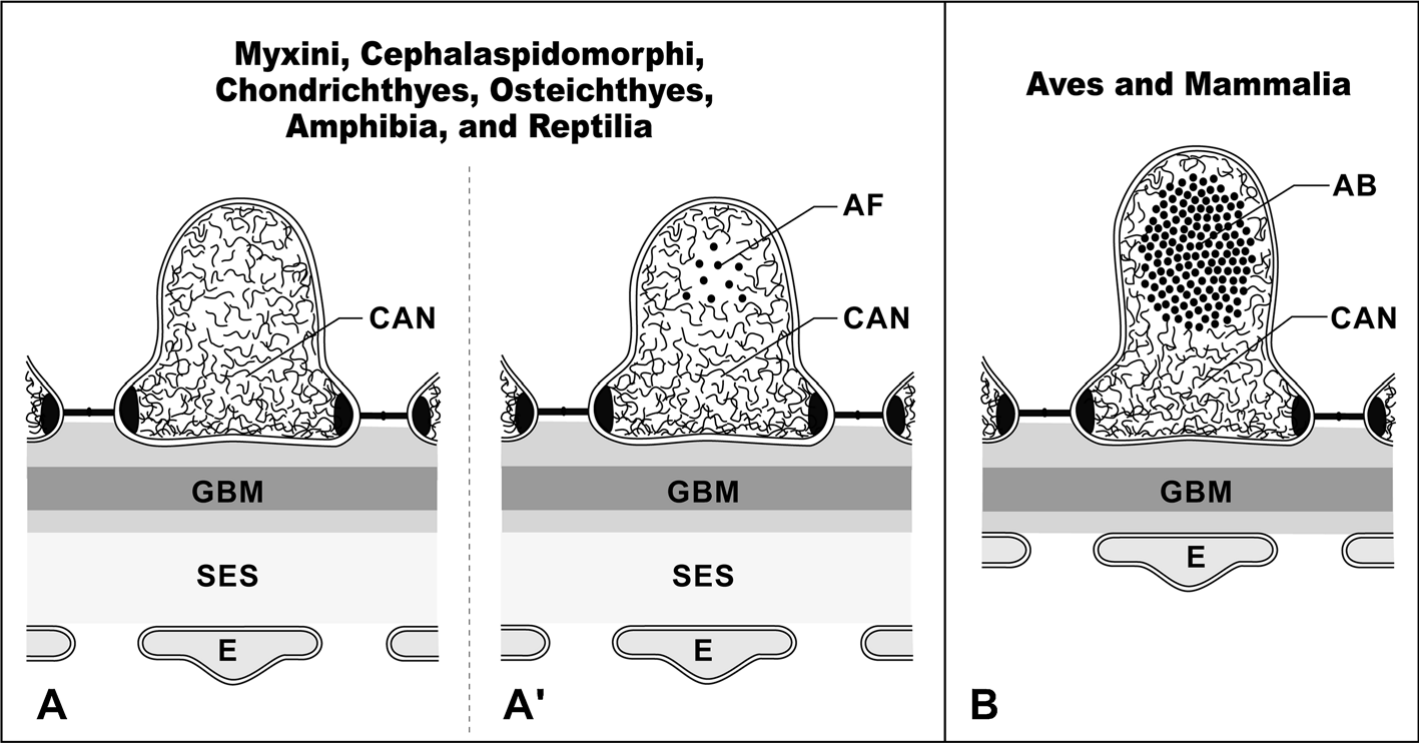
Actin bundle in foot processes. Schematic representations showing actin filament organization of foot processes in vertebrates. The cortical actin network (CAN) predominantly occupies the cytoplasm of the foot processes in vertebrates other than birds and mammals (**a**). The actin fascicle (AF) is also recognized at various frequencies (**a**). In contrast, in birds and mammals, the conspicuous actin bundle (AB) is located in the upper cytoplasm of foot processes above the level of the slit diaphragm, and the cortical actin network occupies the space between the actin bundle and the plasma membrane (**b**).* E* Glomerular endothelial cell,* GBM* glomerular basement membrane,* SES* subendothelial space. The schematic drawing is based on Ichimura et al. (2007)

In birds and mammals, the glomerular capillary wall is exposed to high GCP. Therefore, several mechanical protectors evolved to shield the glomerular capillary wall from the high GCP. One of the mechanical protectors is actin bundles in podocyte foot processes (Figs. 4j, k, 10c) (Ichimura et al. 2003, 2007). These actin bundles are composed of tightly bundled actin filaments located in the foot processes. Unlike the stress fibers, the actin bundles possess no dense body and are not in direct contact with the basal plasma membrane. The actin bundle is linked to the GBM through a cortical actin network, which surrounds the actin bundle, and integrin molecules in the basal plasma membrane, thus contributing to the local strengthening of the GBM. Other vertebrates lack such actin bundles in the foot processes; instead, the cortical actin network is found predominantly in the foot processes (Figs. 4g–i, 10a, b).

#### Actin filament condensations in podocytes

Actin filament condensation is peculiar to mammalian podocytes, and is found in the irregularly shaped thick processes of podocytes, occupying the angle between the adjacent capillary loops (Ichimura et al. 2009). Actin filament condensations link the GBM of adjacent capillary loops, and the linkage stabilizes the high folding pattern of the GBM (Kriz et al. 1995).

#### Disappearance of primary cilium in podocytes

The glomerular podocytes in vertebrates generally exhibit a single primary (non-motile) cilium with 9 + 0 axoneme (Miyoshi 1978; Ojeda et al. 2003; Zuasti et al. 1983). However, in mammals and birds, the primary cilia disappear from the podocytes during glomerular development (Ichimura et al. 2010). Along with the increase of glomerular filtration rate during development, the primary cilia on immature podocytes are subjected to a stronger bending force, and the subsequent influx of calcium ions via the polycystin complex is increased. Therefore the disappearance of primary cilium in podocytes prevents the disturbance of intracellular signal cascade by excessive influx of calcium ions.

## Conclusion

In most of the metazoan taxa, PUPA produce the primary urine by ultrafiltration in filtration epithelial cells (terminal cells and/or podocytes). The basic structure of the filtration site, which consists of filtration slits and slit diaphragms, is highly conserved among metazoans, despite the diversity in overall architecture of the PUPA and filtration epithelial cells. These facts indicate that ultrafiltration is the most effective way to produce primary urine in metazoans and that the basic structure of the filtration site is suitable for the production of primary urine by ultrafiltration.

The protonephridium is regarded as a primitive form of excretory system in metazoans, and the metanephridium as a more advanced form. However, Ruppert and Smith (1988) proposed a comprehensive explanation of the diversity of metazoan PUPA that is consistent with many observations. They proposed that whether animals utilize the protonephridial terminal cells or the metanephridial podocytes as filtration epithelial cells depends on body design, especially body size. They also considered the terminal cells in protonephridium and the podocytes in metanephridium homologous. Thus, the terminal cell-based PUPA can be regarded as an extremely small coelom, which comprises only a terminal cell(s) without squamous mesothelial cells.

According to the above theory, the Bowman’s capsules of mesonephros and metanephros represent a minute coelom, which is created within mesonephros and metanephros independently from the original deuterocoel. This interpretation is partially supported by the fact that both mesothelial cells and podocytes specifically express the transcription factor Wilms’ tumor suppressor 1 (WT1), which plays a crucial role in the development of mesothelium and glomerulus in vertebrates (Perner et al. 2007; Rackley et al. 1993; Serluca 2008). Moreover, common molecular components are utilized to form and maintain the slit diaphragm of the filtration epithelial cells in some metazoans such as vertebrate podocytes (zebrafish, rodent, human), nephrocytes (fruit fly, gastropod) (Kokkinopoulou et al. 2014; Weavers et al. 2009; Zhuang et al. 2009), and terminal cells (planarian) (Thi-Kim Vu et al. 2015). It is thus highly possible that common molecular mechanisms underlying the formation of metazoan PUPA will be discovered in the near future.

## References

[CR1] Balser EJ, Ruppert EE (1990). Structure, ultrastructure, and function of the preoral heart-kidney in *Saccoglossus kowalevskii* (Hemichordata, Enteropneusta) including new data on the stomochord. Acta Zoologica.

[CR2] Baylis C, Brenner BM (1978). The physiologic determinants of glomerular ultrafiltration. Rev Physiol Biochem Pharmacol.

[CR3] Beyenbach KW, Skaer H, Dow JA (2010). The developmental, molecular, and transport biology of Malpighian tubules. Annu Rev Entomol.

[CR4] Boer HH, Sminia T (1976). Sieve structure of slit diaphragms of podocytes and pore cells of gastropod molluscs. Cell Tissue Res.

[CR5] Boute N, Gribouval O, Roselli S (2000). NPHS2, encoding the glomerular protein podocin, is mutated in autosomal recessive steroid-resistant nephrotic syndrome. Nat Genet.

[CR6] Braun EJ, Dantzler WH, Dantzler WH (1997). Vertebrate renal system. Handbook of physiology, section 13. Comparative physiology.

[CR7] Elger M, Sakai T, Kriz W (1998). The vascular pole of the renal glomerulus of rat. Adv Anat Embryol Cell Biol.

[CR8] Farquhar MG, Palade GE (1962). Functional evidence for the existence of a third cell type in the renal glomerulus—phagocytosis of filtration residues by a distinctive” third” cell. J Cell Biol.

[CR9] Fedeles S, Gallagher AR (2013). Cell polarity and cystic kidney disease. Pediatr Nephrol.

[CR10] Gagliardini E, Conti S, Benigni A, Remuzzi G, Remuzzi A (2010). Imaging of the porous ultrastructure of the glomerular epithelial filtration slit. J Am Soc Nephrol.

[CR11] Goodman SH, Cavey MJ (1990). Organization of a phyllobranchiate gill from the green shore crab *Carcinus maenas* (Crustacea, Decapoda). Cell Tissue Res.

[CR12] Ichimura K, Kurihara H, Sakai T (2003). Actin filament organization of foot processes in rat podocytes. J Histochem Cytochem.

[CR13] Ichimura K, Kurihara H, Sakai T (2006). Involvement of mesangial cells expressing alpha-smooth muscle actin during restorative glomerular remodeling in Thy-1.1 nephritis. J Histochem Cytochem.

[CR14] Ichimura K, Kurihara H, Sakai T (2007). Actin filament organization of foot processes in vertebrate glomerular podocytes. Cell Tissue Res.

[CR15] Ichimura K, Stan RV, Kurihara H, Sakai T (2008). Glomerular endothelial cells form diaphragms during development and pathologic conditions. J Am Soc Nephrol.

[CR16] Ichimura K, Kurihara H, Sakai T (2009). Beta-cytoplasmic actin localization in vertebrate glomerular podocytes. Arch Histol Cytol.

[CR17] Ichimura K, Kurihara H, Sakai T (2010). Primary cilia disappear in rat podocytes during glomerular development. Cell Tissue Res.

[CR18] Ichimura K, Fukuyo Y, Nakamura T (2013). Developmental localization of nephrin in zebrafish and medaka pronephric glomerulus. J Histochem Cytochem.

[CR19] Ichimura K, Miyazaki N, Sadayama S (2015). Three-dimensional architecture of podocytes revealed by block-face scanning electron microscopy. Sci Rep.

[CR20] Inokuchi S, Kimura K, Sugaya T (1998). Angiotensin II maintains the structure and function of glomerular mesangium via type 1a receptor. What we have learned from null mutant mice minus the angiotensin II type 1a receptor gene. Virchows Arch.

[CR21] Ishii S (1980). The ultrastructure of the protonephridial flame cell of the freshwater planarian Bdellocephala brunnea. Cell Tissue Res.

[CR22] Jollie M (1973). Chordate morphology.

[CR23] Kestila M, Lenkkeri U, Mannikko M (1998). Positionally cloned gene for a novel glomerular protein–nephrin—is mutated in congenital nephrotic syndrome. Mol Cell.

[CR24] Kieneke A, Ahlrichs WH, Arbizu PM, Bartolomaeus T (2008). Ultrastructure of protonephridia in Xenotrichula carolinensis syltensis and *Chaetonotus maximus* (Gastrotricha: Chaetonotida): comparative evaluation of the gastrotrich excretory organs. Zoomorphology.

[CR25] Kokkinopoulou M, Guler MA, Lieb B, Barbeck M, Ghanaati S, Markl J (2014). 3D-ultrastructure, functions and stress responses of gastropod (*Biomphalaria glabrata*) rhogocytes. PLoS ONE.

[CR26] Kramer-Zucker AG, Wiessner S, Jensen AM, Drummond IA (2005). Organization of the pronephric filtration apparatus in zebrafish requires Nephrin, Podocin and the FERM domain protein Mosaic eyes. Dev Biol.

[CR27] Kriz W, Kaissling B, Alpern RJ, Hebert SC (2007). Structural organization of the mammalian kidney. Seldin and Giebisch’s the kidney—physiology and pathophysiology.

[CR28] Kriz W, Elger M, Mundel P, Lemley KV (1995). Structure-stabilizing forces in the glomerular tuft. J Am Soc Nephrol.

[CR29] Mayer G, Bartolomaeus T (2003). Ultrastructure of the stomochord and the heart–glomerulus complex in *Rhabdopleura compacta* (Pterobranchia): phylogenetic implications. Zoomorphology.

[CR30] McDonald MD, Grosell M (2006). Maintaining osmotic balance with an aglomerular kidney. Comp Biochem Physiol A: Mol Integr Physiol.

[CR31] McKanna JA (1968). Fine structure of the protonephridial system in planaria I. Flame cells. Zeitschrift für Zellforschung und Mikroskopische Anatomie.

[CR32] Miyoshi M (1978). Scanning electron microscopy of the renal corpuscle of the mesonephros in the lamprey, *Entosphenus japonicus* Martens. Cell Tissue Res.

[CR33] Mundel P, Kriz W (1995). Structure and function of podocytes: an update. Anat Embryol.

[CR34] Ojeda JL, Icardo JM, Domezain A (2003). Renal corpuscle of the sturgeon kidney: an ultrastructural, chemical dissection, and lectin-binding study. Anat Rec A Discov Mol Cell Evol Biol.

[CR35] Pavenstadt H, Kriz W, Kretzler M (2003). Cell biology of the glomerular podocyte. Physiol Rev.

[CR36] Perner B, Englert C, Bollig F (2007). The Wilms tumor genes wt1a and wt1b control different steps during formation of the zebrafish pronephros. Dev Biol.

[CR37] Praetorius HA (2015). The primary cilium as sensor of fluid flow: new building blocks to the model. A review in the theme: cell signaling: proteins, pathways and mechanisms. Am J Physiol Cell Physiol.

[CR38] Praetorius HA, Spring KR (2003). The renal cell primary cilium functions as a flow sensor. Curr Opin Nephrol Hypertens.

[CR39] Prosser C, Prosser CL (1973). Circulation of body fluids. Comparative animal physiology.

[CR40] Rackley RR, Flenniken AM, Kuriyan NP, Kessler PM, Stoler MH, Williams BR (1993). Expression of the Wilms’ tumor suppressor gene WT1 during mouse embryogenesis. Cell Growth Differ.

[CR41] Rodewald R, Karnovsky MJ (1974). Porous substructure of the glomerular slit diaphragm in the rat and mouse. J Cell Biol.

[CR42] Ruppert EE (1994). Evolutionary origin of the vertebrate nephron. Am Zool.

[CR43] Ruppert E, Smith P (1988). The functional organization of filtration nephridia. Biol Rev.

[CR44] Ruppert E, Fox R, Barnes R (2003). Invertebrate zoology—a functional evolutionary approach.

[CR45] Sakai T, Kriz W (1987). The structural relationship between mesangial cells and basement membrane of the renal glomerulus. Anat Embryol.

[CR46] Saxen L (1987). Organogenesis of the kidney.

[CR47] Schaffner A, Rodewald R (1978). Filtration barriers in the coelomic sac of the crayfish, *Procambarus clarkii*. J Ultrastruct Res.

[CR48] Seifert G, Rosenberg J (1977). Die Ultrastruktur der Nephrozyten vonPeripatoides leuckarti (Saenger 1869) (Onychophora, Peripatopsidae). Zoomorphologie.

[CR49] Serluca FC (2008). Development of the proepicardial organ in the zebrafish. Dev Biol.

[CR50] Singh SR, Hou SX (2008). Lessons learned about adult kidney stem cells from the malpighian tubules of Drosophila. J Am Soc Nephrol.

[CR51] Takahashi-Iwanaga H (2002). Comparative anatomy of the podocyte: a scanning electron microscopic study. Microsc Res Tech.

[CR52] Takahashi-Iwanaga H (2015) Three-dimensional microanatomy of the pericapillary mesangial tissues in the renal glomerulus: comparative observations in four vertebrate classes. Biomed Res 36(5):331–341 10.2220/biomedres.36.331 26522150

[CR53] Thi-Kim Vu H, Rink JC, McKinney SA et al (2015) Stem cells and fluid flow drive cyst formation in an invertebrate excretory organ. Elife 4. doi:10.7554/eLife.07405 10.7554/eLife.07405 PMC4500094 26057828

[CR54] Turpenniemi TA, Hyvarinen H (1996). Structure and role of the renette cell and caudal glands in the nematode *Sphaerolaimus gracilis* (Monhysterida). J Nematol.

[CR55] Wartiovaara J, Ofverstedt LG, Khoshnoodi J (2004). Nephrin strands contribute to a porous slit diaphragm scaffold as revealed by electron tomography. J Clin Invest.

[CR56] Weavers H, Prieto-Sanchez S, Grawe F (2009). The insect nephrocyte is a podocyte-like cell with a filtration slit diaphragm. Nature.

[CR57] Wilson RA, Webster LA (1974). Protonephridia. Biol Rev Camb Philos Soc.

[CR58] Zhang F, Zhao Y, Chao Y, Muir K, Han Z (2013). Cubilin and amnionless mediate protein reabsorption in Drosophila nephrocytes. J Am Soc Nephrol.

[CR59] Zhang F, Zhao Y, Han Z (2013). An in vivo functional analysis system for renal gene discovery in Drosophila pericardial nephrocytes. J Am Soc Nephrol.

[CR60] Zhuang S, Shao H, Guo F, Trimble R, Pearce E, Abmayr SM (2009). Sns and Kirre, the Drosophila orthologs of Nephrin and Neph1, direct adhesion, fusion and formation of a slit diaphragm-like structure in insect nephrocytes. Development.

[CR61] Zuasti A, Agulleiro B, Hernandez F (1983). Ultrastructure of the kidney of the marine teleost *Sparus auratus*: the renal corpuscle and the tubular nephron. Cell Tissue Res.

